# Effect of Phacoemulsification on Anterior Chamber Angle in Eyes with Medically Uncontrolled Filtered Primary Angle-Closure Glaucoma

**DOI:** 10.1155/2020/8720450

**Published:** 2020-04-21

**Authors:** Chengguo Zuo, Bing Long, Xinxing Guo, Liming Chen, Xing Liu

**Affiliations:** ^1^State Key Laboratory of Ophthalmology, Zhongshan Ophthalmic Center, Sun Yat-Sen University, Guangzhou, China; ^2^The Third Affiliated Hospital, Sun Yat-Sen University, Guangzhou, China; ^3^Wilmer Eye Institute, Johns Hopkins University, Baltimore, MD, USA

## Abstract

**Purpose:**

To evaluate the effect of phacoemulsification and intraocular lens (IOLs) implantation in eyes with medically uncontrolled primary angle-closure glaucoma (PACG) previously treated with trabeculectomy and to quantify the anatomical changes in the anterior chamber angle by ultrasound biomicroscopy (UBM).

**Methods:**

Forty-four eyes of 37 consecutive patients with medically uncontrolled PACG coexisting cataracts with a surgical history of trabeculectomy were included in this study. Each patient underwent phacoemulsification and IOL implantation. Indentation gonioscopy and UBM were performed preoperatively and then again 3 months after surgery. The main outcome measures were best-corrected visual acuity (BCVA), intraocular pressure (IOP), number of antiglaucoma medications and anatomical changes in the anterior chamber angle.

**Results:**

The mean logarithm of the minimum angle of resolution BCVA significantly improved from 0.52 ± 0.30 preoperatively to 0.26 ± 0.23 postoperatively (*p* < 0.001). The mean IOP significantly decreased from 24.33 ± 9.65 mmHg preoperatively to 18.04 ± 7.86 mmHg postoperatively (*p* < 0.05). 001). The median number of antiglaucoma medications decreased from 2 preoperatively to 1 postoperatively (*p* < 0.001). There was no significant difference in the extent of peripheral anterior synechia after the surgery (*p* > 0.05). Some parameters, including anterior central chamber depth, angle opening distance at 500 *μ*m, trabecular-iris angle, and scleral ciliary process angle, were significantly higher after than before surgery (*p* < 0.001). However, the crystalline lens rise was significantly smaller following the surgery (*p* < 0.001).

**Conclusions:**

Phacoemulsification and IOL implantation reduced the IOP and improved vision in eyes with medically uncontrolled filtered PACG. The mechanism underlying the outcomes observed following surgery might be related to the anterior chamber deepening, widened drainage angle, and improved aqueous fluid flow to the trabecular meshwork.

## 1. Introduction

Glaucoma is the leading cause of irreversible blindness worldwide [1]. Primary angle-closure glaucoma (PACG) is an important type of glaucoma that has a high prevalence in Asia and is a large burden in China [2]. Trabeculectomy is a classic treatment for PACG, but traditional trabeculectomy is associated with a high risk of postoperative complications, such as malignant glaucoma, bleb-related infections, and surgical failure [3]. Trabeculectomy can also increase the risk of cataract formation and progression [4]. The treatment of medically uncontrolled PACG after initial trabeculectomy is more complicated.

In recent years, cataract surgery, even clear lens extraction, has become a first-line therapy for PACG. Numerous studies have shown that phacoemulsification and intraocular lens (IOL) implantation can increase the anterior chamber depth (ACD), open the irido-corneal angle, and decrease the intraocular pressure (IOP) [5–8]. However, few studies have evaluated the effect of phacoemulsification on anterior chamber angle in eyes with medically uncontrolled PACG previously treated with trabeculectomy.

In this retrospective study, we quantified the effect of phacoemulsification and intraocular lens (IOL) implantation on anterior chamber angle in eyes with medically uncontrolled filtered primary angle-closure glaucoma by means of indentation gonioscopy and ultrasound biomicroscopy (UBM).

## 2. Methods

### 2.1. Subjects

The study sample was composed of 44 eyes of 37 consecutive patients with medically uncontrolled filtered PACG. After phacoemulsification and IOL implantation, the patients were followed up for at least 6 months (19.2 ± 14.1 months; range: 6–76 months).

The inclusion criteria included the following: (1) PACG with at least 270 degrees of irido-trabecular contact (appositional or synechiae on gonioscopy) and glaucomatous optic neuropathy determined based on optic disc cupping and glaucomatous visual field loss, (2) IOP > 21 mmHg with maximally tolerated medications, (3) no less than 3 months after surgery of initial trabeculectomy, and (4) coexisting cataract. The exclusion criteria were as follows: (1) the presence of any cause of secondary glaucoma, including uveitic glaucoma, neovascular glaucoma, exfoliative glaucoma, and phacomorphic glaucoma; (2) a history of any previous eye surgery except trabeculectomy or more than two trabeculectomy surgeries, (3) previous ocular trauma, and (4) other severe ocular diseases.

Complete success was defined as IOP ≤ 21 mmHg without any glaucoma medications. Qualified success was defined as the same IOP level but with medications. Failure was defined as IOP > 21 mmHg with maximally tolerated medications.

This study was performed at the Glaucoma Department, Zhongshan Ophthalmic Center, Sun Yat-sen University, Guangzhou, China. The study was approved by the Ethics Committee at Zhongshan Ophthalmic Center. All patients provided informed consent in accordance with the Declaration of Helsinki.

### 2.2. Preoperative and Postoperative Evaluation

All subjects underwent a complete ophthalmic examination, including a best-corrected visual acuity (BCVA) test, slit lamp examination, Goldmann applanation tonometry, fundoscopy, and 24-2 Humphrey visual fields (Allergan Humphrey, San Leandro, CA). Contact A-scan biomicroscopy (Nidek US-1800, Japan) was performed to measure the axial length and lens thickness. Visual acuity was measured with a Snellen chart, and the value was converted into the logarithm of the minimum angle of resolution (logMAR). Postoperative values were measured at 1, 3, 6, and 9 months after surgery and every 3 months thereafter until the last visit. A gonioscopic examination was performed with a Goldmann gonioscope in a dark room, and the results were recorded preoperatively and postoperatively at month 3 by the same researcher. Peripheral anterior synechia (PAS) was confirmed when the pigmented trabecular meshwork was not visible on indentation gonioscopy, and appositional angle closure was confirmed when the pigmented part of the trabecular meshwork was not visible on static gonioscopy but visible on indentation gonioscopy. The extent of synechial angle closure was recorded as the clock hour position.

**Figure 1:**
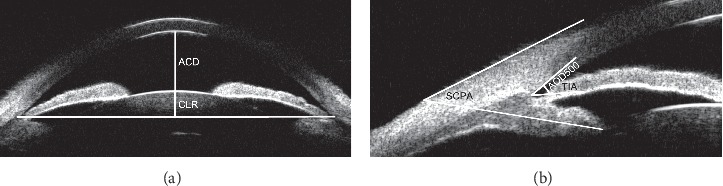
An illustration of the quantitative measurement of the ACD, CLR, AOD500, TIA, and SCPA. (a) ACD: anterior chamber depth; CLR: crystalline lens rise. (b) AOD500: angle opening distance at 500 *μ*m from a scleral spur; TIA: trabecular-iris angle; SCPA: scleral ciliary process angle.

UBM was also performed preoperatively and postoperatively after 3 months with a UBM SW-3200L machine (model SW-3200L; Tianjin Suowei Electronic Technology Co, Ltd., Tianjin, China), which reproduces images with a high axial and lateral resolution of 40 *μ*m. A UBM examination was performed under constant room illumination with the patient supine and fixating on a distant target to control accommodation. Radial scans were performed at the 12, 3, 6, and 9o'clock positions in addition to an axial scan. Standard photographs were selected for the qualitative assessment of the parameters. The scleral spur was defined as the point at which a change in curvature of the inner surface of the angle wall became apparent. This often presented as an inward protrusion of the sclera [9]. After the scleral spur was located, the following variables were measured [10] (Figure 1).

The central ACD was measured as the axial distance from the corneal endothelium to the anterior lens surface.

The angle opening distance at 500 *μ*m (AOD500) was defined as the distance between the posterior corneal surface and the anterior iris surface and was measured as a line running perpendicular from the trabecular meshwork to a point 500 *μ*m from the scleral spur.

The trabecular-iris angle (TIA) was measured as follows: the apex of the triangle was located in the iris recess, while the arms of the angle passed through a point on the trabecular meshwork located 500 *μ*m from the scleral spur and the point lying perpendicular and opposite on the iris.

The scleral ciliary process angle (SCPA) was measured as the angle between a line that was tangential to the scleral surface and a line forming the axis of the ciliary body.

The crystalline lens rise (CLR) was defined as the distance between the anterior pole of the crystalline lens and the perpendicular point on a horizontal line joining the two scleral spurs.

### 2.3. Surgical Procedure

All operations were performed under topical anesthesia by a single surgeon (Liu X). Phacoemulsification and IOL implantation were performed through a 3.2-mm temporal clear cornea incision. A foldable IOL was implanted into the capsular bag after the cortical material was aspirated. Postoperatively, a 4-week course of tapering topical steroids and antibiotics was routinely prescribed. If the IOP remained higher than 21 mmHg after surgery, antiglaucoma eye drops were prescribed.

### 2.4. Statistical Analysis

SPSS software (V. 16.0, USA) was used for statistical analysis. A *p* value of less than 0.05 was considered statistically significant. Preoperative and postoperative BCVA, IOP, and anterior segment parameters were compared using paired Student's *t* test. The preoperative and postoperative number of anti-glaucoma medications and the extent of PAS were compared using the Wilcoxon signed-rank test.

## 3. Results

Forty-four eyes of 37 patients, including 8 male and 29 female subjects, with a mean age of 62.8 ± 7.7 years, were included. The demographic and clinical features of the study participants are presented in Table 1. The mean axial length and lens thickness were 22.25 ± 0.76 mm and 5.21 ± 0.26 mm, respectively.

The mean LogMAR BCVA improved significantly from 0.52 ± 0.30 preoperatively to 0.26 ± 0.23 postoperatively (*p* < 0.001). The mean IOP decreased significantly from 24.33 ± 9.65 mmHg to 18.04 ± 7.86 mmHg (*p* < 0.001). The median number of medications decreased from 2 preoperatively to 1 postoperatively (*p* < 0.001). There was no significant difference in the extent of PAS between before and after the surgery (*p*=0.714) (Table 2). At the last visit, twenty-one out of 44 (47.7%) patients were considered complete successes. Seventeen (38.6%) patients were qualified successes (defined as treated with antiglaucoma medications). The mean IOP decreased by 25.9%, and the total success rate was 86.4% in this group. The IOP of six (13.6%) patients remained out of control even when the maximal tolerated medications were administered and eventually required additional antiglaucoma surgery. Further analysis showed that, in patients with a PAS extent less than or equal to 11 clock hours, the failure rate was 6.7%, while in those with a PAS extent more than 11 clock hours, the failure rate reached 28.6%. The difference between the two groups was significant (*p* < 0.05).

None of the cases developed aqueous misdirection or endophthalmitis. Two patients experienced transient corneal edema after surgery; these complications resolved spontaneously within 1 week postoperatively.

**Figure 2:**
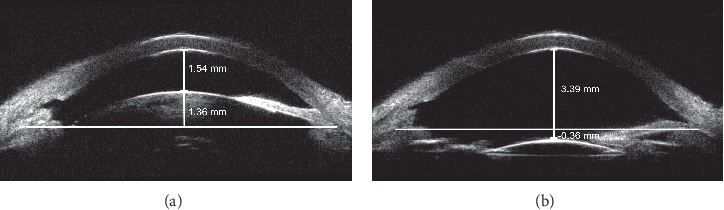
Example of the UBM of a female patient aged 56 years with medically uncontrolled filtered PACG both pre- and posttreatment. (a) Before phacoemulsification and IOL implantation, the ACD and CLR were 1.54 and 1.36 mm, respectively. (b) After surgery, the anterior chamber was significantly deepened. The ACD and CLR were 3.39 and −0.36 mm, respectively.

**Figure 3:**
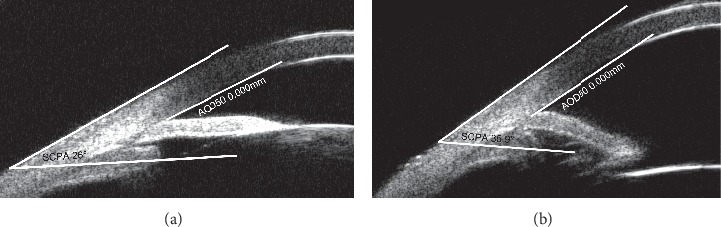
Example of the UBM of the same patient shown in Figure 2 before and after treatment. (a) Preoperative analysis of the nasal angle showed that the SCPA and AOD50 were 26° and 0 mm, respectively. (b) Postoperative analysis of the same angle demonstrated that the SCPA and AOD50 were 35.9° and 0 mm, respectively.

The results of our comparisons between preoperative and postoperative UBM parameters are summarized in Table 3. The parameters demonstrated that the mean ACD, mean AOD500, mean TIA, and mean SCPA were significantly higher after surgery than before surgery (*p* < 0.001), while the mean CLR was significantly lower following the surgery (*p* < 0.001, Figures 2 and 3).

## 4. Discussion

PACG is caused by disorders of the iris, lens, and retrolenticular structures [11]. Phacoemulsification, rather than trabeculectomy, is currently the recommended first-line surgical treatment for PACG. Trabeculectomy should, in general, be considered if the IOP remains too high despite laser and medical treatment. However, in a previous study, the failure rate of primary trabeculectomy in PACG patients was 32% due to postoperative scarring and other complications [12] and was even higher following secondary trabeculectomy. Under these circumstances, cataract surgery can be performed to avoid the complications of filtering surgery and open the anterior chamber space for subsequent possible trabeculectomy or glaucoma drainage valve implantation. Numerous studies have shown that having a thickened and anteriorly positioned lens is a pivotal contributor to the pathogenesis of PACG [13, 14] and that lens extraction could be a first-line treatment in these patients [14]. However, whether this surgery is effective in medically uncontrolled PACG eyes with previous filtration surgeries needs to be confirmed.

In the present study, IOP and the number of medications significantly decreased following phacoemulsification and IOL implantation in filtered PACG eyes. The mean LogMAR BCVA significantly improved from 0.52 ± 0.30 preoperatively to 0.26 ± 0.23 postoperatively, and the mean IOP significantly decreased from 24.33 ± 9.65 mmHg preoperatively to 18.04 ± 7.86 mmHg postoperatively. The median number of medications decreased from 2 preoperatively to 1 postoperatively. These results were consistent with those found in two previous studies conducted in whole filtered PACG patients. Moghimi et al. [15] evaluated 37 previously filtered eyes in 37 PACG patients who underwent phacoemulsification at least 12 months after trabeculectomy. The IOP significantly decreased from 18.16 ± 5.91 mmHg at baseline to 15.37 ± 2.90 mmHg at the final follow-up. The mean number of antiglaucoma medications significantly decreased from 1.81 before to 0.86 after surgery. Yang et al. [16] reported on 47 eyes in 47 PACG patients who had undergone trabeculectomy followed by phacoemulsification at least 3 months later, in whom the IOP significantly decreased from 18.7 ± 6.7 mmHg at baseline to 16.4 ± 2.0 mmHg at the final follow-up. The median number of antiglaucoma medications was also significantly lower at 2 years postoperative. In the present study, patients were followed up for at least 6 months, and our results, when combined with those of previous studies, demonstrate that performing cataract surgery in eyes with filtered PACG effectively reduced IOP and the number of patients requiring antiglaucoma drugs.

Although the mean IOP decreased by 25.9%, the total success rate was only 86.4% in the present study, and this was not as high as the success rate achieved in previous studies conducted in whole filtered PACG patients [15, 16]. The total success rate reported in the previous studies was 97.2% [15] and 89.4% [16]. The reason for our relatively lower success rate may be because more than half of the patients enrolled in previous studies had already achieved complete or qualified success before phacoemulsification. However, in our study, the IOP of all the patients exceeded 21 mmHg even with the preoperative application of maximally tolerated medications. Our results also demonstrate that the failure rate of patients with a PAS less than or equal to 11 clock hours was only 6.7%, which was significantly lower than that achieved in those with PAS of more than 11 clock hours. The reason for the inferior prognosis achieved in our study might be that the range of PAS was too large, making it more difficult to reopen or widen the anterior chamber angle.

The exact mechanism underlying this reduction in IOP is not well understood. Previous studies have assumed that phacoemulsification can eliminate the pupillary blockage and alleviate the angle crowding caused by a thickened and anteriorly positioned lens. Mechanical deepening the anterior chamber with viscoelastic and saline infusion during phacoemulsification may open some PAS. Moghimi et al. [15] found that the average angle width significantly increased from grade 0.31 ± 0.51 preoperatively to grade 2.01 ± 0.73 at one year postoperatively in their patients. These authors also found that ACD significantly increased from 2.21 ± 0.32 mm preoperatively to 2.57 ± 0.30 mm postoperatively. Yang et al. [16] proposed that cataract extraction would relieve apposition, even in patients who undergo synechial, angle closure to lower IOP. However, this proposal lacks objective morphological evidence.

UBM is a very useful and important tool for identifying the characteristics of anterior chamber angles. UBM also allows the quantitative measurement of different anterior segment parameters. To the best of our knowledge, only a few studies have investigated the anatomical changes that occur in the anterior chamber after lens surgery in PACG patients. In this study, we objectively and precisely assessed anterior segment parameters by UBM. Our results reveal that there were significant increases in ACD, AOD500, TIA, and SCPA and that there was a significant decrease in CLR following surgery. Accordingly, although we found that the median PAS extent decreased from 11 to 10 after phacoemulsification, this decrease was not significant. Previous studies have inadequately reported on the anatomic anterior-segment changes that occur in filtered PACG patients [17, 18]. Our results are similar to those achieved in previous investigations conducted in surgery-naive PACG patients. Previous studies have demonstrated that cataract extraction exerts significant effects on anterior chamber anatomy in patients with PACG [19]. While the present study found the change in the extent of PAS was very insignificant after phacoemulsification. It was inconsistent with what was previously reported in literature. In a randomized controlled clinical trial, Man et al. [19] demonstrated that AOD500 and ACD increased after cataract surgery in 26 eyes with PACG and no previous surgery. The mean extent of synechial angle closure was significantly reduced from 272.3° to 253.3° by phacoemulsification [19]. The reason may be that the extent of PAS in our study was too wide to be separated. Another reason may be that, in the present study, the PAS is more tightly adhered due to long-term uncontrollable intraocular pressure and the inflammatory stimulation of previous trabecular surgery. Our findings suggest that changes occurred in the above parameters to deepen the anterior chamber, widen the drainage angle, and improve the access of aqueous fluid flow to the trabecular meshwork. All these factors may contribute to the reduction in IOP observed in eyes with filtered PACG.

However, the limitations of this study should be recognized. The sample size was relatively small, and the follow-up time was not long. These results therefore need to be confirmed in a randomized controlled clinical trial with a longer follow-up period.

## 5. Conclusion

Phacoemulsification is useful in cases of medically uncontrolled filtered PACG, in which it can be performed as the initial procedure of choice. The mechanism underlying the outcomes observed following surgery might be related to the anterior chamber deepening, widened drainage angle, and improved aqueous fluid flow to the trabecular meshwork.

## Figures and Tables

**Table 1:** Demographic and clinical features of study participants.

Characteristics	Value	Range
Eye/patient (*n*)	44/37	48, 79
Mean age (years)	62.8 ± 7.7	
Male/female sex (*n*)	8/29	
Axial length (mm)	22.25 ± 0.76	19.90, 23.48
Lens thickness (mm)	5.21 ± 0.26	4.68, 5.74

**Table 2:** Comparisons between preoperative and postoperative logMAR BCVA, IOP, PAS, and number of medications.

	Mean logMAR BCVA	Mean IOP (mmHg)	PAS (median clock hours)	Median no. of medications
Before operation	0.52 ± 0.30	24.33 ± 9.65	11 (2, 12)	2 (1, 4)
After operation	0.26 ± 0.23	18.04 ± 7.86	10 (1, 12)	1 (0, 4)
*p*	<0.001	<0.001	0.714	<0.001

**Table 3:** Comparisons between preoperative and postoperative UBM parameters.

	Preoperation	Postoperation	*p*
Mean ACD (mm)	1.76 ± 0.29	3.73 ± 0.32	<0.001
Mean AOD500 (mm)	0.03 ± 0.04	0.08 ± 0.08	<0.001
Mean TIA (°)	3.13 ± 4.52	8.69 ± 8.20	<0.001
Mean SCPA (°)	30.34 ± 4.37	38.30 ± 4.08	<0.001
Mean CLR (mm)	1.10 ± 0.27	-0.76 ± 0.25	<0.001

## Data Availability

The data used to support the findings of this study are included within the article.

## References

[1] Flaxman S. R., Bourne R. R. A., Resnikoff S. (2017). Global causes of blindness and distance vision impairment 1990–2020: a systematic review and meta-analysis. *Lancet Glob Health*.

[2] Liang Y. B., Wang N. L., Rong S. S., Thomas R. (2015). Initial treatment for primary angle-closure glaucoma in China. *Journal of Glaucoma*.

[3] Luebke J., Neuburger M., Jordan J. F. (2019). Bleb-related infections and long-term follow-up after trabeculectomy. *International Ophthalmology*.

[4] Rotchford A. P., King A. J. (2010). Moving the goal posts. *Ophthalmology*.

[5] Shams P. N., Foster P. J. (2012). Clinical outcomes after lens extraction for visually significant cataract in eyes with primary angle closure. *Journal of Glaucoma*.

[6] Tham C. C. Y., Kwong Y. Y. Y., Baig N., Leung D. Y. L., Li F. C. H., Lam D. S. C. (2013). Phacoemulsification versus trabeculectomy in medically uncontrolled chronic angle-closure glaucoma without cataract. *Ophthalmology*.

[7] Chen P. P., Lin S. C., Junk A. K., Radhakrishnan S., Singh K., Chen T. C. (2015). The effect of phacoemulsification on intraocular pressure in glaucoma patients. *Ophthalmology*.

[8] Moghimi S., Latifi G., ZandVakil N. (2015). Phacoemulsification versus combined phacoemulsification and viscogonioplasty in primary angle-closure glaucoma. *Journal of Glaucoma*.

[9] Seager F. E., Wang J., Arora K. S., Quigley H. A. (2014). The effect of scleral spur identification methods on structural measurements by anterior segment optical coherence tomography. *Journal of Glaucoma*.

[10] Henzan I. M., Tomidokoro A., Uejo C. (2010). Ultrasound biomicroscopic configurations of the anterior ocular segment in a population-based study. *Ophthalmology*.

[11] Weinreb R. N., Aung T., Medeiros F. A. (2014). The pathophysiology and treatment of glaucoma. *Journal of the American Medical Association*.

[12] Zhang H., Tang G., Liu J. (2016). Effects of phacoemulsification combined with goniosynechialysis on primary angle-closure glaucoma. *Journal of Glaucoma*.

[13] Tarongoy P., Ho C. L., Walton D. S. (2009). Angle-closure glaucoma: the role of the lens in the pathogenesis, prevention, and treatment. *Survey of Ophthalmology*.

[14] Azuara-Blanco A., Burr J., Ramsay C. (2016). Effectiveness of early lens extraction for the treatment of primary angle-closure glaucoma (EAGLE): a randomised controlled trial. *The Lancet*.

[15] Moghimi S., Latifi G., Amini H. (2013). Cataract surgery in eyes with filtered primary angle closure glaucoma. *Journal of Ophthalmic and Vision Research*.

[16] Yang W. H., Han Q., Chen S., Yan H. (2016). The effect of phacoemulsification on intraocular pressure in eyes with primary angle closure glaucoma after trabeculectomy. *Zhonghua Yan Ke Za Zhi*.

[17] Tham C. C. Y., Leung D. Y. L., Kwong Y. Y. Y., Li F. C. H., Lai J. S. M., Lam D. S. C. (2010). Effects of phacoemulsification versus combined phaco-trabeculectomy on drainage angle status in primary angle closure glaucoma (PACG). *Journal of Glaucoma*.

[18] Shao T., Hong J., Xu J., Le Q., Wang J., Qian S. (2015). Anterior chamber angle Assessment by anterior-segment optical coherence tomography after phacoemulsification with or without goniosynechialysis in patients with primary angle closure glaucoma. *Journal of Glaucoma*.

[19] Man X., Chan N. C. Y., Baig N. (2015). Anatomical effects of clear lens extraction by phacoemulsification versus trabeculectomy on anterior chamber drainage angle in primary angle-closure glaucoma (PACG) patients. *Graefe’s Archive for Clinical and Experimental Ophthalmology*.

